# A pilot study and histological examination of xenograft and autogenous bone combinations with i-PRF in guided bone regeneration

**DOI:** 10.34172/japid.025.3543

**Published:** 2025-06-28

**Authors:** Saurabh Kamat, Senthil Murugan M, Edlyn Rodrigues, Jochima Eudora Cota, Vikas Dhupar, Francis Akkara

**Affiliations:** ^1^Department of Oral & Maxillofacial Surgery, Goa Dental College and Hospital, Goa , India; ^2^Department of Oral & Maxillofacial Surgery, Saveetha Dental College and Hospital, Tamil Nadu, India; ^3^Department of Oral Pathology, Goa Dental College and Hospital, Goa, India

**Keywords:** Autografts, Bone regeneration, Dental implant, Platelet-rich fibrin

## Abstract

**Background.:**

This study evaluated the impact of different regenerative biomaterial combinations on bone quality and implant stability in guided bone regeneration (GBR).

**Methods.:**

A pilot study was conducted from September 2020 to October 2023 to compare the quality of bone regeneration and implant stability following GBR using three composite graft combinations. Forty-seven patients participated in the study in three experimental groups: group A (deproteinized cancellous bovine bone [xenograft] with injectable platelet-rich fibrin [i-PRF]), group B (xenograft with autogenous bone graft in a 2:1 ratio with i-PRF), and group C (xenograft with autogenous bone graft in a 1:1 ratio with i-PRF). The implant stability quotient (ISQ) was measured at the time of implant placement. Crestal bone biopsy procedures were performed.

**Results.:**

The study found that group C, using a 1:1 ratio of xenograft and autogenous graft with i-PRF, achieved the highest new bone formation (65.83%) and demonstrated moderately high vascularization and osteoclastic activity, indicative of good remodeling potential. ISQ measurements for all groups indicated good primary stability of implants, ranging from 55 to 65 at the time of placement.

**Conclusion.:**

Combining xenograft with autogenous graft in a 1:1 ratio, along with i-PRF, yielded optimal outcomes for new bone formation in GBR procedures. However, further research is needed to address the limitations associated with i-PRF, such as lack of rigidity and faster degradation, to enhance its application in GBR procedures.

## Introduction

 Guided bone regeneration (GBR) is a cornerstone technique in implant dentistry, using barrier membranes to promote selective bone growth in areas of tissue defects near dental implants.^1,2^ By preventing the ingrowth of epithelial and connective tissue cells, GBR fosters bone regeneration while minimizing periodontal infections.^3,4^ Moreover, GBR serves as a well-documented procedure for selective bone formation to regenerate lost alveolar bone anatomy by preventing the ingression of epithelial and connective tissue cells with the help of the cell-occlusive membrane.^5-7^ While autogenous bone grafts are considered the gold standard due to their biocompatibility and osteoinductive properties, the discomfort and morbidity associated with harvesting from a separate surgical site have driven the search for alternative strategies.

 The frequent need for augmenting bone before placing implants, especially in the posterior areas of the upper or lower jaw, has led to the development of bone substitutes.^8^ Clinical and histological evidence supports the efficacy of various biomaterials, including autogenous bone chips, allografts, and xenografts, in addressing bone augmentation needs. Although autologous grafts are biologically safe, they present a myriad of challenges, including the need for additional donor sites and postoperative complications.^9,10^ As alternatives, xenogeneic or alloplastic materials have emerged, meeting criteria such as biocompatibility, osteoconductivity, and resorbability. Notably, deproteinized bovine bone mineral (DBBM), exemplified by BioOss^TM^, exhibits both biocompatibility and osteoconductive properties, proving effective in diverse procedures, encompassing sinus floor augmentation, preservation of the alveolar ridge, and treatment of peri-implant defects.^9^ To optimize the beneficial qualities of each biomaterial, combinations of these materials have been proposed, offering a comprehensive approach to enhancing bone regeneration outcomes in dental implant procedures.^11^

 Research has been done to improve wound healing and bone regeneration in dental procedures by combining bone substitutes with growth factors.^12,13^ Platelet-rich fibrin (PRF), derived from the patient’s peripheral blood, contains platelets, leukocytes, and growth factors.^14^ Solid and liquid forms of PRF offer flexibility in application.^15^ While systematic reviews highlight the positive effects of PRF in dental surgery, particularly in soft tissue and periodontal treatment, its benefits in bone regeneration lack strong evidence.^16^

 Despite the effectiveness of GBR, the discomfort associated with harvesting autogenous bone grafts prompts the exploration of alternative strategies. This study addresses the need for novel approaches by investigating three regenerative biomaterial combinations in GBR. While various biomaterials have shown promise, including autogenous bone chips, allografts, and xenografts, a consensus on the most effective graft combination remains lacking. This research aims to fill this gap by evaluating the impact of different biomaterial combinations on bone quality and implant stability, contributing new insights to the field.

## Methods

 A comprehensive study was conducted to compare the quality of bone regeneration and assess implant stability following GBR using three composite graft combinations from September 2020 to October 2023. Forty-seven patients participated in the study, with 88 implants placed.

 All the patients provided written informed consent. The study received ethical approval from the Dental College & Hospital Institutional Ethics Committee (Ref. No.: GDCH/IEC/III-2020 (11)-PROV).

###  Inclusion criteria

- Patients aged 21‒75 years
- Patients undergoing delayed implant placements with a maximum of 4 implant threads at the crestal region and requiring GBR

###  Exclusion criteria

- Patients with any systemic debilitating conditions such as uncontrolled diabetes mellitus or hypertension
- Patients undergoing immediate implant placements

###  Study groups

 Patients were randomly assigned to one of the three experimental groups using a computer-generated randomization process. The allocation sequence was concealed in opaque, sealed envelopes, which were opened by the surgical team immediately before the procedure. Randomization ensured even distribution across the groups while accounting for the variability in defect morphology.

 Group A (n = 14; 25 implants): a composite graft of xenograft and i-PRF

 Group B (n = 20; 39 implants): a composite graft of xenograft and autogenous graft (2:1 ratio) bound together with i-PRF

 Group C (n = 13; 24 implants): a composite graft of xenograft and autogenous graft (1:1 ratio) bound together using i-PRF

 The rationale for selecting the 2:1 and 1:1 ratio in groups B and C was based on prior evidence suggesting improved osteoconductive properties with higher autogenous bone proportions, balanced against the potential for donor site morbidity.

###  Surgical procedure

 Before the procedures, the patients received stringent antibiotic prophylaxis (amoxicillin, 500 mg, and clavulanic acid, 125 mg - Augmentin, provided by GlaxoSmithKline Malta Ltd., Malta).^17^ The antibiotic prophylaxis was initiated one hour before the surgery and continued at regular intervals postoperatively for 120 hours. Implant osteotomies were performed with precision, and implant stability quotient (ISQ) measurements were taken using the Penguin RFA unit (Integration Diagnostics, Sweden AB) at the time of implant placement. Buccal bone decortication was performed to optimize the regenerative process.

###  Bone graft and membrane use

 A resorbable membrane (Bio-Gide^®^, Geistlich, Wolhusen, Switzerland) was used. Graft combinations were prepared as follows: xenograft and i-PRF for group A, xenograft, autogenous graft (2:1 ratio), and i-PRF for group B, and xenograft, autogenous graft (1:1 ratio), and i-PRF for group C. Graft volumes were measured using the technique described by Delvin et al.^18^ The i-PRF was freshly prepared using patients’ own blood and centrifuged at 700 rpm for 3 minutes in test tubes without anticoagulants. Autogenous bone scrapes were obtained using a Buser scraper (HuFriedy Group) either from the apical areas of the same surgical site or from a donor site (external oblique ridge).

 Following the surgical procedure, the patients underwent a healing phase that lasted approximately 4 months. Re-entry was performed for abutment placement, with an indentation mark made at the implant site to guide sample collection. Bone samples (an average diameter of 2.5 mm and a length of 10 mm) were obtained using a #21 blade from implant beds. The samples were fixed in 10% buffered formalin, and tissue decalcification was achieved using a solution containing formic acid, formaldehyde, and deionized water (Decalcifier-Fixative Gooding Stewart, Bio-Optica Milano s.p.a). Sections of 4‒7-μm thickness were prepared and stained with Hematoxylin and Eosin (H&E) for histological examination under light microscopy at × 400 magnification coupled with Image Access software (Imagic, Glattbrugg, Switzerland).

###  Histological evaluation

 Randomly chosen fields were evaluated for new bone volume and residual graft material, and the presence of vascularization, osteoblasts/osteoclasts, and granulocytes was assessed. Vascularization was determined by observing new vessel formation around and within graft material and newly formed bone. The surgeon performed all implant placements, and all study personnel and patients were aware of group assignments. Histomorphometric variables measured bone vitality, remodeling, and maturity, including new bone volume and residual graft material. The histometric analysis was conducted by an examiner trained in histology, using a 400 × magnification and a calibrated grid eyepiece.

###  Statistical analysis

 Statistical analysis was performed using SPSS, where data were entered into Microsoft Excel. Descriptive statistics were applied. Comparative analyses between study groups involved Fisher’s exact test and one-way ANOVA, followed by post hoc Tukey tests. A significance threshold of *P* < 0.05 was considered statistically significant.

## Results

**Figure 1:**
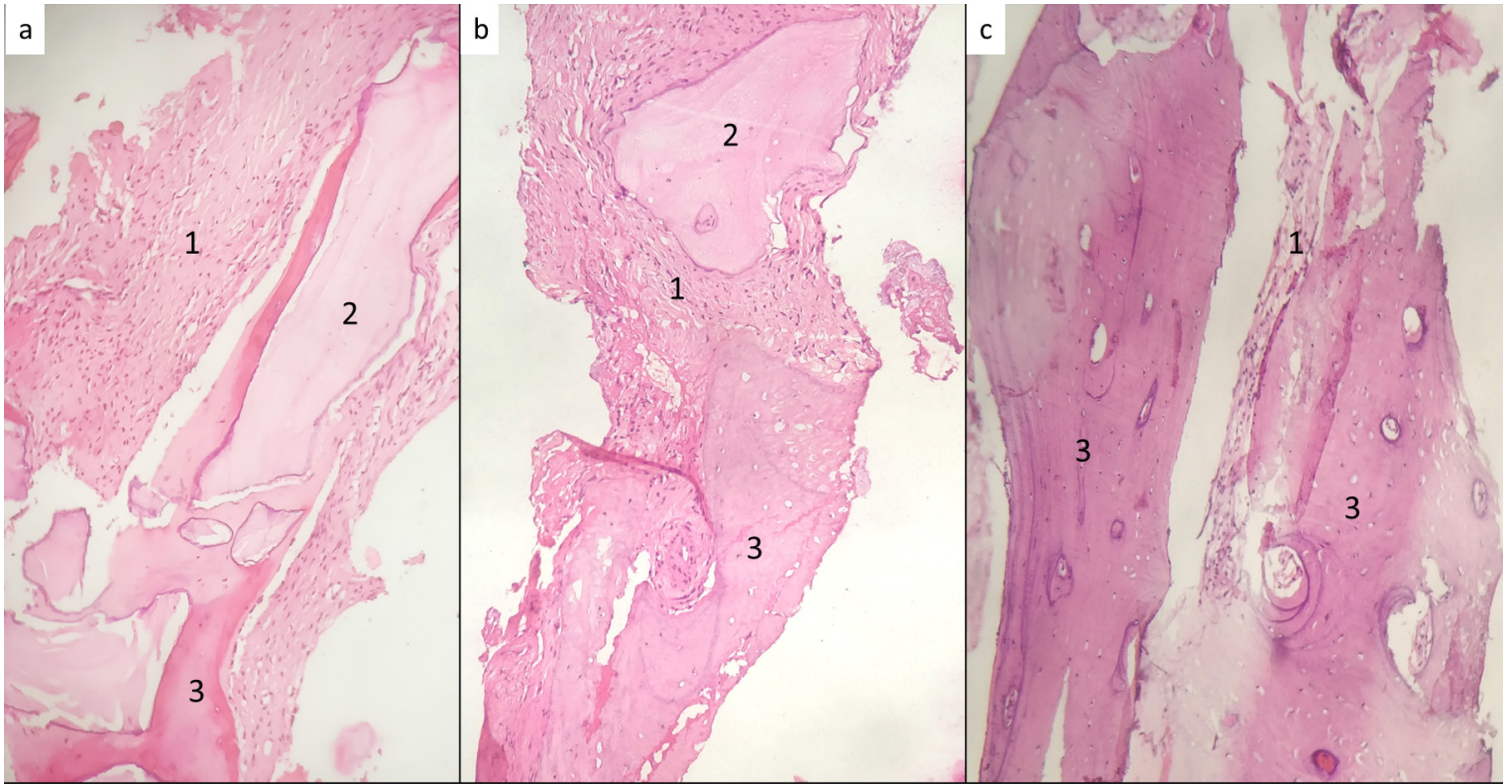
Histological sections showing (a) xenograft with i-PRF, (b) xenograft with autogenous bone graft (2:1) and i-PRF, and (c) xenograft with autogenous bone graft (1:1) and i-PRF. Staining: H&E; Magnification: 400 × . 1: Connective tissue; 2: Bio-Oss; 3: Bone

Table 1 presents the individual responses within each group. Table 2 compares all the groups in terms of new bone formation and residual graft material. Substantial variations were observed in new bone formation and the percentages of residual graft material. Group C exhibited the highest new bone formation (65.83%), significantly outperforming groups A (19.00%) and B (39.62%; P < 0.001). Additionally, group A had the highest residual graft material percentage (57.20%), significantly surpassing groups B (35.26%) and C (20.65%; *P* < 0.001; Figure 1).

 Pairwise comparisons between study groups using post hoc Tukey tests are tabulated in Table 3. However, in terms of the percentage of new bone formation, all pairwise comparisons revealed statistically significant differences (*P* < 0.001). Group A exhibited significantly lower new bone formation compared to groups B and C. In contrast, group B also had significantly lower new bone formation than group C. Group A exhibited significantly more residual graft material than groups B and C (*P* < 0.001), with group C having the least.

Table 4 presents a comprehensive analysis of vascularization and osteoclastic activity across the study groups. Significant differences were observed in vascularization (*P* = 0.001), with the percentage of a low degree of vascularization ( + /-) decreasing from 100.0% in group A to 66.7% in group C. In the moderately high degree of vascularization ( + + ) category, group C stood out with a rate of 33.3%. For osteoclastic activity, significant differences were found with similar percentages in the mild osteoclastic activity ( + ) category (72.0%, 76.9%, and 75.0% for groups A, B, and C, respectively). Group C did not exhibit low osteoclastic activity ( + /-), while groups A and B showed 28.0% and 23.1% of low osteoclastic activity, respectively. Only group C exhibited moderately high osteoclastic activity ( + + ), probably suggestive of remodeling. ISQ measurements for all groups indicated good primary stability of implants, ranging from 55 to 65 at the time of placement and above 75 at second-stage surgery (secondary stability).

**Table 1:** Patient data in all study groups

**Groups**	**Patient No.**	**Tooth No.**	**New bone formation (%)**	**Residual graft material (%)**	**Vascularization**	**Osteoclastic activity**
Group A	1	5	20	55	+	+
		6	15	65	+	+
		7	20	50	+	+
	2	21	10	70	+	+
		22	15	60	+	+
	3	9	25	50	+	+
	4	8	35	40	+	+ /-
	5	26	15	65	+	+
		27	15	65	+	+
		28	20	55	+	+
	6	3	25	45	+	+
		4	20	60	+	+
	7	14	25	65	+	+ /-
		15	20	50	+	+ /-
	8	22	10	75	+	+
		24	15	70	+	+
		26	10	65	+	+
	9	27	15	55	+	+ /-
	10	12	15	55	+	+
	11	7	20	60	+	+
	12	12	25	50	+	+ /-
		13	25	55	+	+ /-
		14	20	50	+	+ /-
	13	29	15	40	+	+
	14	28	25	60	+	+
Group B	1	10	35	45	+	+
	2	12	35	40	+	+
		13	45	30	+	+
	3	6	50	25	+	+
		7	40	25	+	+
	4	27	45	25	+	+
		28	40	30	+	+
	5	20	35	55	+ +	+ /-
		21	30	55	+ +	+ /-
		22	35	40	+ +	+ /-
	6	19	30	45	+	+
		20	35	40	+	+
	7	8	40	30	+	+
	8	30	40	25	+	+
	9	22	45	30	+	+ /-
		24	45	25	+	+ /-
		26	45	40	+	+ /-
	10	10	30	40	+	+
		11	30	45	+	+
		12	40	30	+	+
	11	7	35	40	+	+
	12	10	35	40	+	+
		11	40	30	+	+
		12	35	35	+	+
	13	12	40	35	+	+
		13	45	35	+	+
	14	28	55	30	+	+
		29	60	25	+	+
	15	19	50	35	+	+ /-
		20	45	30	+	+ /-
		21	35	35	+	+ /-
	16	27	40	35	+	+
		24	40	30	+	+
	17	4	40	35	+	+
		6	35	40	+	+
		7	40	40	+	+
	18	13	30	35	+	+
	19	5	35	40	+	+
	20	9	40	30	+	+
Group C	1	6	80	10	+	+
		8	75	15	+	+
	2	10	65	25	+ +	+
		12	70	15	+ +	+
	3	19	65	15	+	+
	4	3	60	25	+	+
		4	70	30	+	+
	5	19	65	20	+ +	+ +
		20	65	25	+ +	+ +
		21	60	30	+ +	+ +
	6	6	55	25	+ +	+ +
		4	65	20	+ +	+ +
		3	65	25	+ +	+ +
	7	27	60	20	+	+
		25	75	10	+	+
	8	5	70	15	+	+
	9	12	65	10	+	+
	10	29	55	35	+	+
		30	65	2 5	+	+
	11	8	70	20	+	+
		6	55	25	+	+
	12	21	65	25	+	+
		19	70	20	+	+
	13	27	70	15	+	+

+ Mild (10% to 30% of the microscopic field); + + Moderately high (30% to 60% of the microscopic field); + /- Low ( < 10% of the microscopic field); - Not present.

**Table 2:** Comparison of new bone formation and residual graft material between the study groups

**Variable**	**Study groups**	**N**	**Mean**	**SD**	**Min**	**Max**	**ANOVA**	
							**F**	***P*** ** value**
New bone formation (%)	A	25	19.00	5.951	10	35	322.70	< 0.001*
	B	39	39.62	6.823	30	60		
	C	24	65.83	6.370	55	80		
Residual graft material (%)	A	25	57.20	9.138	40	75	132.23	< 0.001*
	B	39	35.26	7.604	25	55		
	C	23	20.65	6.793	10	35		

*P < 0.05 Statistically significant; *P* > 0.05 Non-significant.

**Table 3:** Pairwise comparison of new bone formation and residual graft material between the study groups

**Variable**	**Comparison group 1**	**Comparison group 2**	**Mean difference**	**SD**	***P *** **value**	**95% Confidence interval**	
						**Lower bound**	**Upper bound**
New bone formation (%)	A	B	-20.62	1.66	< 0.001*	-24.57	-16.66
		C	-46.83	1.85	< 0.001*	-51.24	-42.43
	B	C	-26.22	1.68	< 0.001*	-30.22	-22.22
Residual graft material (%)	A	B	21.94	2.02	< 0.001*	17.13	26.76
		C	36.55	2.28	< 0.001*	31.12	41.98
	B	C	14.60	2.07	< 0.001*	9.66	19.55

*P < 0.05 Statistically significant; *P* > 0.05 Non-significant.

**Table 4:** Post hoc Tukey tests comparing histological parameters between the groups

**Variable**	**Grading of histological activity**	**Study groups**			**Total**	**Fisher’s exact test**
		**A**	**B**	**C**		***P*** ** value**
Vascularization	+	25	36	16	77	0.001*
		100.0%	92.3%	66.7%	87.5%	
	+ +	0	3	8	11	
		0.0%	7.7%	33.3%	12.5%	
Osteoclastic Activity	+	18	30	18	66	< 0.001*
		72.0%	76.9%	75.0%	75.0%	
	+ /-	7	9	0	16	
		28.0%	23.1%	0.0%	18.2%	
	+ +	0	0	6	6	
		0.0%	0.0%	25.0%	6.8%	

*P < 0.05 Statistically significant, *P* > 0.05 Non-significant. + mild (10% to 30% of the microscopic field); + + moderately high (30% to 60% of the microscopic field); + /- low ( < 10% of the microscopic field); - not present

## Discussion

 Our pilot study demonstrated that the optimal outcome for new bone formation (65.83%) was achieved through the combination of xenograft and autogenous bone graft in a 1:1 ratio with i-PRF. This finding is consistent with the traditional use of autogenous bone graft, considered the gold standard due to its cellular and molecular elements that support osteogenesis.^19^ Allogenic grafts have shown promise, offering mechanical properties comparable to those of autologous bone despite lacking viable cells and retaining the collagenous matrix and natural bone proteins.^20^ However, they also lack important properties needed for a GBR scaffold, such as mechanical strength and volume maintenance due to slow resorption.

 Contrary to our findings, a clinical study using allogenic graft reported lower mean values of new bone in the combination group (allografts with autograft) compared to the group with allogeneic graft alone (35% and 39%).^21^ However, no significant difference between the groups was observed, possibly due to variations in the ratio of autogenous bone grafts used, which was 30%^21^ and 50%.^22^

 Regarding graft resorption rates, our findings showed that group C exhibited a moderately high degree of osteoclastic activity. Reports indicate a wide range of autogenous bone graft resorption rates (12% to 80%).^23^ In contrast to our study, two clinical studies using allogenic graft reported higher rates of resorption in the combined group compared to allogeneic bone graft alone, without significant differences.^22,24^

 Furthermore, deproteinized cancellous bovine bone with i-PRF showed a significant 57.20% residual graft material, indicating a slower resorption rate compared to xenografts with autogenous bone graft in a 2:1 ratio. Graft proportions have been a subject of discussion in GBR, with different studies using a different autogenous graft to allogenic graft ratios, such as 50/50%^22^ and 30/70%,^21^ highlighting the lack of agreement in the field. Histological analysis from various studies has presented mixed results regarding new bone formation when combining autogenous and allogeneic bone grafts.

 Finally, group C in our study demonstrated the highest degree of vascularization ( + + ). The moderately high osteoclastic activity in group C indicates good remodeling and, thus, a higher potential for faster replacement of the xenogeneic scaffold with vital new bone.

 In our study, all the groups used i-PRF. Notably, two clinical studies^25,26^ exclusively using PRF for maxillary sinus augmentation demonstrated significant bone gain. Specifically, one case report in a 59-year-old patient showed dense bone-like tissue formation around implants, accompanied by evidence of osteocytes and osteoblasts.^25^ However, when compared to other materials such as hydroxyapatite^27^ and autogenous bone grafting,^28^ PRF did not show significant advantages in promoting osteogenesis. Although PRF may enhance osteogenesis, its limitations, including lack of rigidity and faster degradation,^29^ underscore the need for further research to improve its application in dental procedures.

 The study’s limitations include the lack of information on modifying factors for osseointegration and GBR success, such as smoking history and a history of periodontitis. Additionally, the study did not address potential confounding variables such as the presence of systemic debilitating conditions like uncontrolled diabetes mellitus or hypertension, etc. Furthermore, while the study evaluated three regenerative biomaterial combinations in GBR, it did not explore other potential combinations or variations in surgical techniques that could affect outcomes. Therefore, the findings of the study should be interpreted within the context of these limitations, and future research should aim to address these gaps.

## Conclusion

 This pilot study demonstrated that combining xenograft with autogenous bone in a 1:1 ratio and i-PRF resulted in superior bone regeneration in GBR. These findings support the use of balanced graft compositions to enhance biological and mechanical outcomes. Further studies are needed to optimize PRF-based protocols for clinical use.

## Competing Interests

 The authors declare that they have no competing interests.

## Consent for Publication

 Not applicable.

## Data Availability Statement

 All data generated and analyzed during this study are included in this published article.

## Ethical Approval

 Ethical approval was obtained from the Dental College & Hospital Institutional Ethics Committee (Ref. No.: GDCH/IEC/III-2020 (11)-PROV).
